# Adaptation of *Escherichia coli* to Long-Term Serial Passage in Complex Medium: Evidence of Parallel Evolution

**DOI:** 10.1128/mSystems.00192-16

**Published:** 2017-03-07

**Authors:** Karin E. Kram, Christopher Geiger, Wazim Mohammed Ismail, Heewook Lee, Haixu Tang, Patricia L. Foster, Steven E. Finkel

**Affiliations:** a Department of Biology, California State University, Dominguez Hills, Carson, California, USA; b Molecular and Computational Biology Section, Department of Biological Sciences, University of Southern California, Los Angeles, California, USA; c Department of Biology, Indiana University, Bloomington, Indiana, USA; University of British Columbia

**Keywords:** adaptive evolution, laboratory evolution, long-term stationary phase, survival

## Abstract

Experimental evolution of bacterial populations in the laboratory has led to identification of several themes, including parallel evolution of populations adapting to carbon starvation, heat stress, and pH stress. However, most of these experiments study growth in defined and/or constant environments. We hypothesized that while there would likely continue to be parallelism in more complex and changing environments, there would also be more variation in what types of mutations would benefit the cells. In order to test our hypothesis, we serially passaged *Escherichia coli* in a complex medium (Luria-Bertani broth) throughout the five phases of bacterial growth. This passaging scheme allowed cells to experience a wide variety of stresses, including nutrient limitation, oxidative stress, and pH variation, and therefore allowed them to adapt to several conditions. After every ~30 generations of growth, for a total of ~300 generations, we compared both the growth phenotypes and genotypes of aged populations to the parent population. After as few as 30 generations, populations exhibit changes in growth phenotype and accumulate potentially adaptive mutations. There were many genes with mutant alleles in different populations, indicating potential parallel evolution. We examined 8 of these alleles by constructing the point mutations in the parental genetic background and competed those cells with the parent population; five of these alleles were found to be adaptive. The variety and swiftness of adaptive mutations arising in the populations indicate that the cells are adapting to a complex set of stresses, while the parallel nature of several of the mutations indicates that this behavior may be generalized to bacterial evolution.

**IMPORTANCE** With a growing body of work directed toward understanding the mechanisms of evolution using experimental systems, it is crucial to decipher what effects the experimental setup has on the outcome. If the goal of experimental laboratory evolution is to elucidate underlying evolutionary mechanisms and trends, these must be demonstrated in a variety of systems and environments. Here, we perform experimental evolution in a complex medium allowing the cells to transition through all five phases of growth, including death phase and long-term stationary phase. We show that the swiftness of selection and the specific targets of adaptive evolution are different in this system compared to others. We also observe parallel evolution where different mutations in the same genes are under positive natural selection. Together, these data show that while some outcomes of microbial evolution experiments may be generalizable, many outcomes will be environment or system specific.

## Abstract (precis)

With a growing body of work directed toward understanding the mechanisms of evolution using experimental systems, it is crucial to decipher what effects the experimental setup has on the outcome. If the goal of experimental laboratory evolution is to elucidate underlying evolutionary mechanisms and trends, these must be demonstrated in a variety of systems and environments. Here, we perform experimental evolution in a complex medium allowing the cells to transition through all five phases of growth, including death phase and long-term stationary phase. We show that the swiftness of selection and the specific targets of adaptive evolution are different in this system compared to others. We also observe parallel evolution where different mutations in the same genes are under positive natural selection. Together, these data show that while some outcomes of microbial evolution experiments may be generalizable, many outcomes will be environment or system specific.

## INTRODUCTION

Adaptation of *Escherichia coli* to laboratory settings has been occurring since the first clinical isolate was taken into the laboratory in 1922 (1). Recent studies have specifically probed how *E. coli* becomes laboratory adapted (or domesticated) (2) as well as how *E. coli* adapts to specific challenges, for instance, nutrient stress (3–7), acid stress (8), or antibiotic stress (9, 10). These studies have allowed researchers to gain insights into the molecular mechanisms of evolution on a practical time scale (2, 5, 11–15). To date, these experimental evolution systems generally use defined minimal medium (4) during serial passage and/or constant conditions in chemostats (2, 3) and therefore may not fully represent the complexity of the adaptive landscapes that *E. coli* may experience in heterogeneous environments (16).

The broad metabolic capacity of *E. coli* allows for growth in a variety of environments in the laboratory, and it can survive in batch culture for days, weeks, or even years without the addition of nutrients (17–20). The typical laboratory life cycle in long-term batch culture consists of five phases, the first three of which are well characterized and commonly studied in the laboratory: lag phase, when cells physiologically adapt to new conditions; log or exponential phase, during which cells have a constant generation time and are presumably under very-low-stress to no-stress conditions; and stationary phase, when cells reach their peak viable cell count, which remains steady for hours to days. The fourth and fifth phases are usually reached during long-term laboratory growth in batch culture. Death phase follows stationary phase, when viable cell counts decrease by >99%. After death phase, the population may enter the fifth phase, “long-term stationary phase” (LTSP). This phase is similar to stationary phase in that the total number of cells remains roughly constant, although at a lower concentration than in stationary phase. However, this phase is distinct from stationary phase in that the relatively constant density is accompanied by significant shifts in subpopulations within the culture, which likely correlate with changing, heterogeneous conditions (17, 19, 21, 22). Survival of cells better suited to their particular environment during LTSP leads to selection of beneficial mutant alleles (18–20, 22, 23), referred to as growth-advantage-in-stationary-phase (GASP) mutations (17, 22, 24). These LTSP-adapted mutants can outcompete parent cells in fresh medium, reflecting adaptive evolution within these populations.

Here, we show that adaptation can occur in relatively few generations if populations are allowed to enter LTSP before passage. We found that after as few as 30 generations (three long-term serial passages) in rich medium, the LTSP survival profiles of the populations had changed, and these cells were better adapted to their environment. This is rapid compared to other examples of experimental evolution in which fitness effects are usually observed after several thousand generations (5, 8, 25). Using whole-genome resequencing of adapted populations, we identified many potential adaptive mutations. There were several instances where different mutations appeared in the same gene in parallel passaged populations, giving potential evidence of parallel evolution and an indication that these genes may be important for adaptation. We show that mutations found in the genes *cytR*, *sspA*, and *tolC* confer a growth advantage during competition with the wild-type (WT) parent, while other mutations identified do not. We believe that with this method of long-term adaptation, we (i) demonstrate how quickly populations of *E. coli* can adapt when presented with multiple selective pressures (nutrient limitation, oxidative stress, and pH variation), along with the heterogeneity that exists during LTSP in complex medium, and (ii) identify physiological processes or metabolic regulatory pathways that are potentially more broadly important for adaptation and evolution.

## RESULTS

### Long-term serially passaged *E. coli* adapts to LTSP.

**FIG 1:**
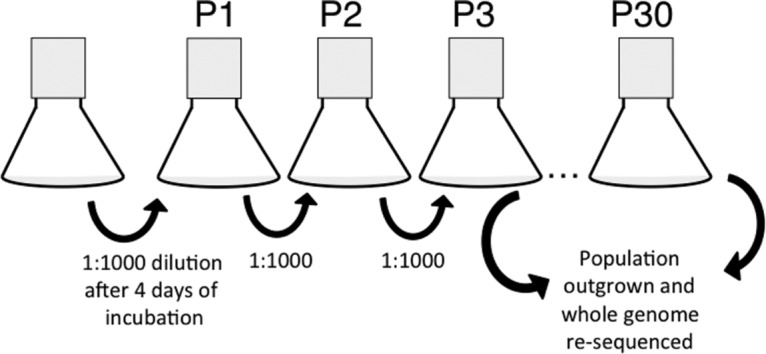
Diagram of passage scheme. The diagram presents the growth scheme that was used to isolate populations. An overnight culture was inoculated into three 125-ml flasks with 12.5 ml of LB at a 1:1,000 dilution. This flask was then incubated with shaking at 200 rpm at 37°C for 4 days. Cells were then passaged into fresh medium at a dilution of 1:1,000. At every passage, CFU per milliliter were counted by serial dilution. Every third passage, 100 μl of cells was saved in glycerol stocks at −80°C. These stocks were then outgrown for growth curves and whole-genome resequencing.

We set out to determine if allowing a population of *E. coli* to incubate through all five phases of the laboratory life cycle would result in the selection of mutants with altered growth phenotypes. In order to accomplish this, parallel cultures were initiated from the same culture of *E. coli* K-12 MG1655-lineage strain PFM2 (26). For our long-term serial passaging scheme, we incubated the cells in the rich medium LB in flasks with shaking for 4 days, a time at which cells are at their lowest density since lag phase, and then serially passaged the cells into fresh medium at a dilution of 1:1,000 (vol/vol), resulting in ~10 generations of growth per passage. We performed 30 of these passages (~300 generations total) in triplicate and after every third passage (~30 generations) saved 1% of the population in frozen stocks (Fig. 1). This serial passaging scheme is distinct from more traditional daily serial passage, where cells are allowed only to enter early stationary phase; therefore, we refer to the scheme here as “long-term serial passage” experiments.

**FIG 2:**
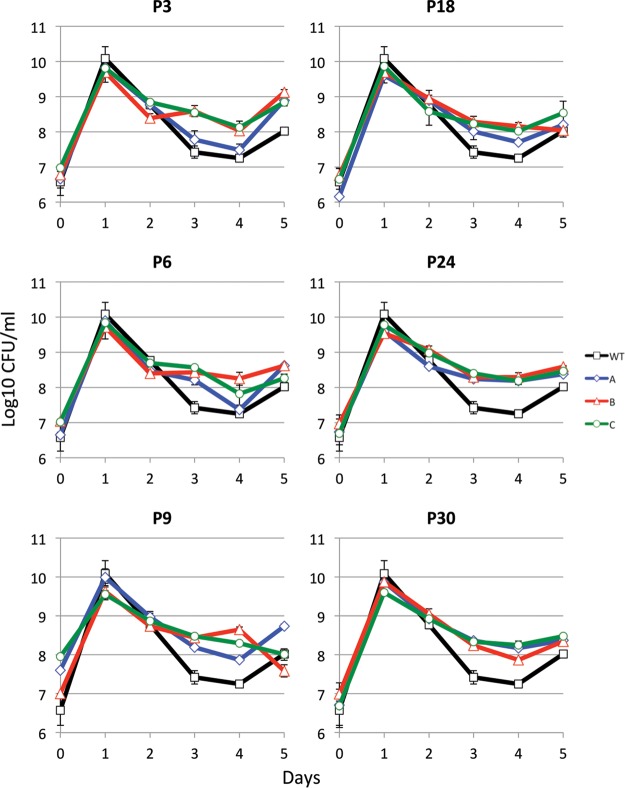
Outgrowth of passaged strains. After saving, populations from each passage were grown in fresh LB medium into LTSP. Each panel represents the growth for 5 days of population A (blue diamonds), population B (red triangles), and population C (green circles) compared to growth of the parent strain (black squares). Each panel is labeled with the passage from which the aged populations were grown. Lines represent the average from two technical replicates, and error bars represent the standard deviation between the two replicates.

Growth phenotypes of passaged cells were determined by monitoring viable counts daily for 5 days, in order to observe not only the growth dynamics during the passage period but recovery as well (Fig. 2). For the parental strain, overnight growth reaches ~5 × 10^9^ CFU/ml on day 1 and then drops more than 100-fold to ~2 × 10^7^ CFU/ml by day 4 of incubation, recovering approximately 10-fold to 2 × 10^8^ CFU/ml by day 5; we refer to this period from 3 to 5 days of growth as the “dip.” In all three passaged populations, long-term growth phenotypes differed (with a *P* value of ≤0.025) from the parent strain as early as passage 3 (P3), indicating that in as few as 30 generations the populations were adapting to the novel LTSP environment. The time points at which the passaged population differs significantly from unaged cells during incubation are listed in Table S1 in the supplemental material. While the apparent length of lag time and log-phase generation time remained similar in all passaged strains (no significant *P* values after only 1 day of incubation), a significant change was observed regarding the timing of and cell density at which populations exit death phase. Long-term serially passaged cells exited death phase earlier at a higher cell density (~10^8^ CFU/ml) after only 2 or 3 days of incubation compared to a viable count of ~10^8^ CFU/ml after 3 or 4 days of incubation for the parental strains. We begin to see these changes as early as passage 3 (Fig. 2).

10.1128/mSystems.00192-16.1 TABLE S1  Time points with significant *P* values (*P* ≤ 0.025) compared to unaged cells. Download TABLE S1, PDF file, 0.03 MB. Copyright © 2017 Kram et al. 2017 Kram et al. This content is distributed under the terms of the Creative Commons Attribution 4.0 International license.

In comparing populations from the last passage (P30) of all three populations to the wild-type parent, it is clear that all three passaged populations exit death phase at an approximately 10-fold-higher density of ~10^8^ CFU/ml as opposed to 10^7^ CFU/ml (Fig. 2). Interestingly, the three passaged populations have adapted in similar but subtly different ways. Populations A, B, and C have densities very similar to the parent strain on the first day of death phase of ~10^9^ CFU/ml. However, population B continues into death phase until day 4, with recovery on day 5. Populations A and C, on the other hand, seem to exit death phase earlier, on day 3. These results indicate that while all three passaged populations have gained mutations that leave them better adapted to long-term growth in LB, the mutations are likely different in each population, leading to different phenotypes.

### Genomic population dynamics during long-term stationary phase.

**FIG 3:**
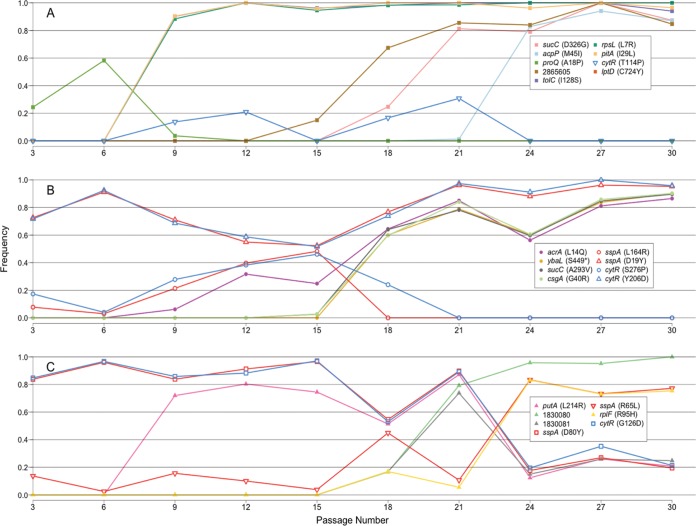
Mutations from passaged populations. Line graph of alleles found in all three populations in each passage that are at some point present in the population above a frequency of 0.3. Frequency was calculated by dividing the number of reads with a nonreference allele by the total number of reads for that nucleotide.

In order to determine what mutations in each population, and at each passage, may lead to the adaptive phenotypes, we performed whole-genome resequencing on cells harvested after every third passage in all three populations. Since there are likely many genotypes present in these cultures at any given time, samples of the entire population were sequenced, instead of single clones picked from each population. This allowed us to identify all of the alleles present above a threshold frequency in each population. Our threshold for calling a mutation was its appearance on each DNA strand at least 3 times; with read depths averaging ~300× for each base sequenced, this procedure results in the detection of mutations present at a threshold of at least 1% of the total population. In total, we identified 213 unique point mutations among all three passaged populations. All mutations are listed in Table S2 and shown in Fig. S1, along with allele frequencies for each population per passage. Identified loci were most frequently polymorphic in the population, with both the parent and mutant alleles represented. However, 13 mutations reached a frequency of 100% in a population for at least some time during the passaging scheme. One hundred four (48.8%) of the mutations were present at only a single time point, and 183 (85.9%) of the loci were never present above 30% in frequency. The highest-frequency alleles are shown in Table 1 and Fig. 3.

10.1128/mSystems.00192-16.2 TABLE S2  SNPs in each population at each time point. Download TABLE S2, XLSX file, 0.1 MB. Copyright © 2017 Kram et al. 2017 Kram et al. This content is distributed under the terms of the Creative Commons Attribution 4.0 International license.

10.1128/mSystems.00192-16.5 FIG S1  Mutations from passaged populations. Line graph of alleles found in all three populations in each passage. Frequency was calculated by dividing the number of reads with a nonreference allele by the total number of reads for that nucleotide. Download FIG S1, PDF file, 3.2 MB. Copyright © 2017 Kram et al. 2017 Kram et al. This content is distributed under the terms of the Creative Commons Attribution 4.0 International license.

**TABLE 1:** High-frequency mutations^a^

Gene	Population	Passage	Genome position	Nucleotide in sequence:		Max frequency	Amino acid in sequence:		Function
				Reference	Mutant		WT	Mutant	
NC	C	30	1830080	G	A	1.00			Upstream of *astC* and *xthA*
NC	C	21	1830081	C	A	0.74			Upstream of *astC* and *xthA*
NC	A	27	2865605	A	G	1.00			Upstream of *rpoS*
*acpP*	A	27	1150972	G	A	0.94	M	I	Acyl carrier protein
*acrA*	B	30	484803	A	T	0.86	L	Q	TolC-AcrAB multidrug efflux pump
*csgA*	B	30	1103787	G	C	0.90	G	R	Curlin
*cytR*	B	15	4121654	A	G	0.46	S	P	DNA utilization negative regulator
*cytR*	B	27	4121864	A	C	1.00	Y	D	DNA utilization negative regulator
*cytR*	C	15	4122103	C	T	0.97	G	D	DNA utilization negative regulator
*cytR*	A	21	4122140	T	G	0.31	T	P	DNA utilization negative regulator
*lptD*	A	27	54939	C	T	1.00	C	Y	Lipopolysaccharide transporter
*pitA*	A	27	3635749	A	C	1.00	I	L	P_i_ transporter
*proQ*	A	6	1913507	C	G	0.58	A	P	Proline transport
*putA*	C	21	1077465	A	C	0.87	L	R	Proline catabolism
*rplF*	C	30	3443879	C	T	0.75	R	H	50S ribosomal component
*rpsL*	A	30	3472555	A	C	1.00	L	R	30S ribosomal component
*sspA*	B	15	3374952	A	C	0.48	L	R	Stringent starvation protein A
*sspA*	C	15	3375205	C	A	0.97	D	Y	Stringent starvation protein A
*sspA*	C	24	3375249	C	A	0.83	R	L	Stringent starvation protein A
*sspA*	B	27	3375388	C	A	0.96	D	Y	Stringent starvation protein A
*sucC*	B	30	763114	C	T	0.90	A	V	Succinyl-CoA synthetase
*sucC*	A	27	763213	A	G	1.00	D	G	Succinyl CoA synthetase
*tolC*	A	27	3176519	T	G	1.00	I	S	TolC-AcrAB multidrug efflux pump
*ybaL*	B	30	501117	G	T	0.90	S	*	Predicted P_i_ transporter

a NC, noncoding. The asterisk represents a stop codon/nonsense mutation.

Surprisingly, 40 of the 213 (18.8%) mutant alleles were present in more than one culture, suggesting that they may have been present during outgrowth of the stock culture and therefore present at initiation of all three cultures. However, ~70% of these mutations (Table S2) were present at very low frequency. While 12 mutations were present above 30% in one population, in a second population they were present at only low frequency (less than 15% with one exception) and often at only one time point (Table S3) and were not detected in the third population. While these data show that the shared mutations may not be significant to the long-term survival of the evolving populations, this high number of mutations stemming from a single outgrowth demonstrates how quickly diversity can be generated.

10.1128/mSystems.00192-16.3 TABLE S3  SNPs found in multiple cultures. Download TABLE S3, XLSX file, 0.1 MB. Copyright © 2017 Kram et al. 2017 Kram et al. This content is distributed under the terms of the Creative Commons Attribution 4.0 International license.

In fact, we can calculate the expected amount of diversity generated from one outgrowth (~10 generations): if we assume that in a 5-ml culture of *E. coli* in LB there are ~10^10^ cells, and the *E. coli* genome is 4.6 × 10^6^ bp, there are ~4.6 × 10^16^ total bp in the culture. The basal mutation frequency in these cultures is ~1 × 10^−10^ mutations/bp replicated (26), which means that there are ~4.6 × 10^6^ mutations in these 4.6 × 10^16^ bp. Since the *E. coli* genome is 4.6 × 10^6^ bp, this suggests that every (nonlethal) mutation may already be present in the population at least once. Therefore, it is impossible to initiate an experiment with no diversity already present in the culture at inoculation densities above ~1 × 10^4^ CFU/ml. Further, this means that all the diversity needed for selection may be generated in the initial outgrowth of this experiment and then selected during each subsequent growth cycle and passage. However, it is equally likely that new variants are generated during each passage.

Several genes or pathways acquired different mutations in separate passaged populations: *cytR* (regulator of nucleoside transport and utilization) (27), *pitA* and *ybaL* (encoding demonstrated [28] and predicted [29] P_i_ transport proteins, respectively), *proQ* and *putA* (proline transport [30] and proline dehydrogenase and transcription factor [31, 32], respectively), *sspA* (stringent response protein) (33), *sucC* (β subunit of succinyl coenzyme A [CoA] synthetase) (34), and *acrA* and *tolC* (TolC-AcrAB multidrug efflux transport system) (35) (Fig. 3; Table 1). Using the multiple alleles of *tolC* as an example, *tolC*(I128S) is present by P9 in population A at a frequency of 90% and remains at 90% to 100% frequency through P30, indicating that it is likely under positive selection. *tolC*(Y415D) is present in population B, starting with passage 6 at a frequency of 20% and increasing to ~30% in P9 but then decreasing to <10% after that. These data may indicate that there is positive selection for one *tolC* allele and not the other or perhaps suggest that in populations B and C (where there are several other low-frequency alleles), other mutations provide a greater adaptive advantage than the *tolC* mutants; further testing is required to distinguish between these possibilities (see below).

In each population, we can begin to see the dynamics of mutations that were occurring during passage. Since we did not sequence individual clones, we cannot determine which mutations are present on the same chromosome. However, if two or more mutations are present in the population at a frequency of greater than 50%, this would indicate cosegregation of mutant alleles in at least a subset of cells. For example, by P6 (60 generations) in population A (Fig. 3A), ~50% of the population has the *proQ*(A18P) mutation; however, by P9 (another 30 generations), this population of cells is almost completely supplanted by cells with three mutations: *tolC*(I128S), *pitA*(I29L), or *rpsL*. It is likely that the vast majority of, if not all, cells in the population have all three of these mutations, because they are each present at such a high frequency. Starting at P15, a subset of this population acquires a fourth mutation in the intergenic region 149 bp upstream of *rpoS* and then acquires a *sucC* mutation by P18 and an *acpP* mutation by P24. By the time of the last passage (P30), it is likely that the majority of cells in the population have acquired all six mutations. We know that some combination of these mutations is likely responsible for the adaptive phenotype that we see with passaged strains (Fig. 2), but further analysis was needed to identify precisely which mutations confer the adaptive phenotype and if any epistatic effects are relevant.

We were particularly interested in the mutations found in the *cytR* gene because CytR, in combination with cyclic AMP receptor protein (CRP), can repress utilization of nucleosides (27). One could imagine that a derepression of this activity might lead to a fitness advantage over wild-type cells in LTSP, because those cells could use nucleic acids, which are likely present at high concentrations after death phase, as a nutrient source (36, 37). Further, unique mutant alleles of *cytR* were detected in all three passaged populations, and in population B, there are two alleles present at a frequency of at least 50% at some point. *cytR*(S276P) is present at 17% by passage 3, increases to 50% by passage 15, and then decreases through passage 21 until it is no longer present in the population. Simultaneously, another allele, *cytR*(Y206D), was present at 72% by passage 3, slightly decreased during P9 to P15 [the same passages during which *cytR*(S276P) increased], and then increased again until it was present at >90% of the population by passage 21 (Fig. 3B).

### Individual point mutations in *cytR*, *sspA*, and *tolC* confer an advantage in long-term cultures.

**FIG 4:**
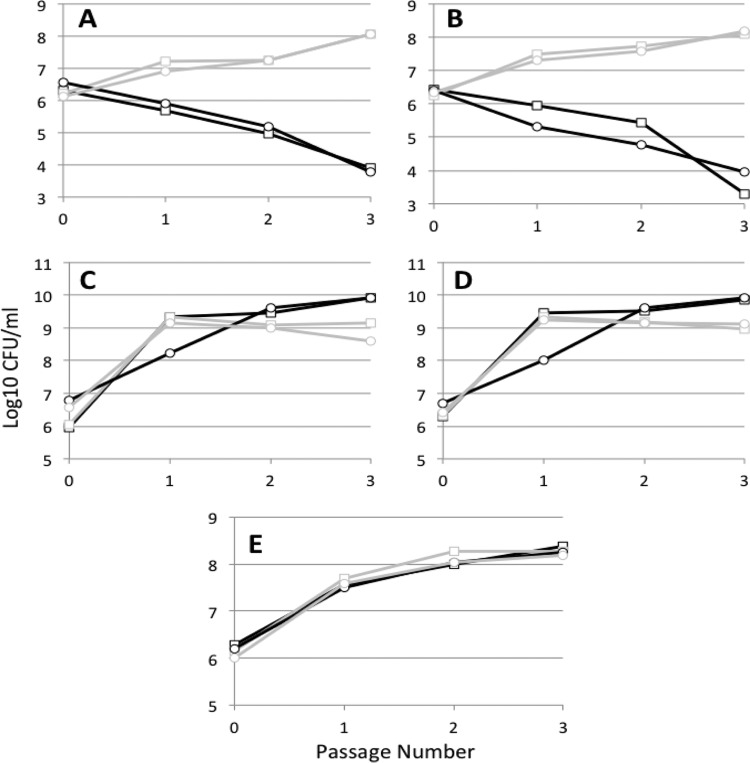
*cytR* mutant versus parent strain competition. Competitions are shown between WT (black) and *cytR*(S276P) (gray) (A and C), WT (black) and *cytR*(Y206D) (gray) (B and D), or *cytR*(S276P) (black) and *cytR*(Y206D) (gray) (E) strains during passages performed every 4 days (A, B, and E) or every 1 day (C and D). “0” indicates the time of inoculation, and the passage number refers to the number of times that the population has been allowed to age and then diluted. Data are shown for two replicate competitions. A Student *t* test was performed at the endpoint of each experiment described above. For panels A, B, and D, *P* values were significant (≤0.01), whereas for panel C the *P* value was 0.06 and for panel E the *P* value was 0.22.

Since the two *cytR* alleles in population B seem to be in competition with each other in the population (Fig. 3B), we wished to determine if they conferred a competitive advantage in competition with either the parental strain or each other. Isogenic strains with these mutant alleles were constructed by site-directed mutagenesis (38). Each *cytR* mutant was competed with the parent in the same long-term serial passage scheme in which the mutations were initially isolated (Fig. 4A and B). Even after one passage, corresponding to 4 days of incubation, the *cytR* mutants have a strong selective advantage over wild-type cells with both *cytR* mutants present at 10-fold-higher viable counts than the wild-type parent. By the third passage, the *cytR* mutant cells outnumber wild-type parents by ~10,000:1. When competed against each other in the long-term (4-day) serial passage scheme, the two mutant strains show no fitness differences (Fig. 4E), indicating that *cytR*(S276P) might have lost out to *cytR*(Y206D) in population B due to stochastic effects. Alternatively, the *cytR*(Y206D) allele may have been present on the chromosome with other mutations that modulated that particular strain’s fitness.

Due to the strong advantage conferred by both mutant *cytR* alleles, we were curious as to why *E. coli* MG1655 has not developed mutations at this locus more frequently over the almost 100 years that it has been cultured in the laboratory and almost 70 years since it has been grown in LB. To address this, we performed more traditional serial passage competitions where cultures were passaged daily instead of every 4 days. This is likely more representative of how laboratory strains of *E. coli* are generally treated. Interestingly, after 3 passages performed every day, the parent strain has reached a density ~10-fold higher than both *cytR* mutants, indicating that wild-type cells have an advantage in this passaging scheme (Fig. 4C and D). Therefore, the advantage of the *cytR* mutants is expressed only in long-term stationary-phase cultures; those same mutations lead to a disadvantage during standard stationary-phase incubation. These data also suggest that daily serial passage in LB would lead to very different adaptive phenotypes than our long-term serial passage scheme.

Site-directed mutants of both *sspA* alleles and, to a lesser degree, the *tolC*(I128S) allele produced results similar to the *cytR* alleles in that the mutant cells have an advantage over wild-type cells [~1 × 10^4^-fold higher for *sspA*(L164R), ~1 × 10^6^-fold higher for *sspA*(D19Y), and ~100-fold higher for *tolC*(I28S) on passage 2] in the 4-day passaging scheme but not in the 1-day passaging scheme [WT is ~10-fold higher than *sspA*(L164R), ~2 to 10-fold higher than *sspA*(D19Y), and ~10-fold higher than *tolC*(I28S) mutants, respectively] (Fig. S2). However, the mutations in *putA*, *pitA*, and *sucC* do not confer any advantage (data not shown), indicating that either they need to be present with another mutation to confer an advantage or they do not make a difference to cells and might have been present by chance. *In silico* studies have suggested that when the environment varies on the same time scale as mutation occurrence, as it likely does in our system, nonbeneficial mutations are just as likely, or even sometimes more likely, to become fixed in a population than beneficial mutations (39). Therefore, in the population A example above, the *tolC* mutation might be the only important mutation in this adapted culture, while the other mutations may be hitchhikers, playing no role in the adaptive phenotype, or they may add to the fitness of the cells epistatically. Experiments are ongoing to identify the epistatic relationships of these mutations.

10.1128/mSystems.00192-16.6 FIG S2  *sspA* and *tolC* mutants versus parent strain competition. Competitions are shown between WT (black) and the mutant strain (gray; see title) during passages performed every 4 days (left column) or every 1 day (right column). “0” indicates the time of inoculation, and the passage number refers to the number of times that the population has been allowed to age and then diluted. Data are shown for two replicate competitions. Download FIG S2, JPG file, 0.1 MB. Copyright © 2017 Kram et al. 2017 Kram et al. This content is distributed under the terms of the Creative Commons Attribution 4.0 International license.

## DISCUSSION

We have shown that in as few as 30 generations we can select for cells that are better adapted to a long-term stationary-phase passaging scheme than their wild-type parents and that after ~300 generations the strains have multiple mutations that may confer the adaptive phenotype. Further, we show that throughout the passaging scheme there are various genotypes that result in the adaptive phenotype, which often contain unique mutations in the same genes, indicating possible parallel evolution. While the evolved genotypes do not delay entry into death phase, they appear to allow cells to exit death phase earlier and at a higher cell density, promoting long-term survival. Competition of cells with point mutations against unaged populations shows that several of the individual mutations identified confer a competitive advantage on parent cells if the populations are allowed to enter death phase and LTSP but not if the cells are passaged during early stationary phase, indicating that these mutations are specifically adaptive in LTSP.

In several other experimental evolution systems, it can take thousands of generations to observe competitive advantage phenotypes (5, 8, 25); here, we observe adaptive mutations in as few as 30 generations. This difference may be due to several features of the experimental design. (i) The adaptive landscape in this experiment likely leads to stronger selection. Because these cells are in a rich, complex medium, and cells are allowed to traverse all five laboratory growth phases, including death phase and LTSP, they are likely experiencing higher levels and a wider variety of stresses than those observed in, for example, minimal medium with low glucose levels. The wide variety of stresses that cells experience, coupled with detrital nutrients released after death phase, may lead to the competitive phenotypes observed here. (ii) Because we are using a rich medium, cells reach higher maximum population sizes (~10^10^ CFU/ml versus the 10^7^ CFU/ml usually observed in low-carbon-source medium). This naturally results in more mutations occurring in each culture upon which selection can act. Further, because we passage the cells at their lowest cell density (10^7^ cells) and at a dilution of 1:1,000, we actually have a narrower bottleneck than in other experiments (3, 5), where often the population is at its largest just before the bottleneck (40). Because of this, we would expect that the sample being passaged would have lower genetic diversity, as opposed to the higher genetic diversity presumed in the work of Wahl and Gerrish (40), meaning that for a mutant cell to be included during passage, it must have been present in the population at relatively high frequency. This passaging scheme also means that there are many more generations between bottlenecks (from ~10^4^ CFU/ml to ~10^10^ CFU/ml is ~20 generations, compared to ~7 generations in 1:100-diluted minimal medium passages). This may lead to the fixation of more mutations (41). Therefore, the system explored here is likely to select for the mutations present at the highest frequencies.

The observed patterns of selection allow us to identify not only what specific genes may be important for adaptation to LTSP in LB but also how these populations are changing over time. To our knowledge, this is the first example of tracking adaptive populations in long-term stationary-phase cultures through all five phases of growth at the molecular genetic level, as opposed to shorter passaging intervals as seen previously (4, 6, 8, 12, 13, 15, 42). All three passaged populations have different paths to their adaptive phenotypes, and while there are several alleles that seem likely to confer an advantage, there are myriad ways and combinations to achieve that population structure. Further, each of the genes identified with adaptive mutations normally has an effect on multiple genes or pathways. CytR is a direct regulator of several operons (27), SspA regulates gene expression at least partially through H-NS activity (43), and mutations in TolC can alter expression of stress response proteins (44). These data suggest that adaptive mutations may be more likely in genes that have pleiotropic effects. Our data support this previously suggested hypothesis (6, 12) and may indicate underlying themes in the evolution of microbes. Further, preliminary whole-transcriptome data suggest that a large number of genes are differentially expressed between aged and unaged populations, including upregulation of several genes in the CytR regulon in aged cells, indicating that *cytR* mutations may in fact lead to differential expression of genes in adapted cells (unpublished observation).

Our data show that adaptive evolution in complex environments may be due to a limited number of genetic pathways, indicating parallel evolution in multiple cultures. Using only three cultures with relatively few generations, we observe many mutations, a small number of which contribute positively to the population. These data indicate that even in a complex, heterogeneous environment, there may be relatively few pathways that lead to strong adaptive phenotypes.

## MATERIALS AND METHODS

### Bacterial strains, growth conditions, and viable cell counts.

An *E. coli* K-12 lineage strain, MG1655 derivative PFM2 (26), was used in this study. The strain was initially grown from a single colony and saved as a frozen stock after one overnight incubation. Three parallel cultures were initiated by transferring cells directly from a frozen 20% glycerol stock into 5 ml of Luria-Bertani (Lennox) medium (LB) (Difco) in an 18- by 150-mm borosilicate test tube and incubated overnight with aeration in a TC-7 rolling drum (New Brunswick Scientific, Edison, NJ) at 37°C. Cells were then diluted into LB at 1:1,000 (vol/vol) to initiate experiments. Cultures were incubated either in test tubes as described above or in 12.5 ml (10% volume) in 125-ml Erlenmeyer flasks on a shaking platform (200 rpm).

Long-term serial passages were performed every 4 days by diluting the culture 1:1,000 in 12.5 ml sterile LB medium in 125-ml Erlenmeyer flasks. Daily serial passages were performed similarly but with passages every 24 h instead of every 4 days. Subsequent growth curves were performed in test tubes. One hundred microliters (0.8%) of the cultures was saved in 20% glycerol stocks every three passages. Cell growth and survival were monitored as described previously (45, 46). Briefly, 10 μl of cells was removed from the 5-ml culture and serially diluted into 90-μl spots of sterile LB. Ten microliters of the dilutions was then spotted on LB plates, up to 8 spots in a row. Individual colonies within one spot were then counted, and CFU per milliliter was calculated by multiplying the dilution factor by the number of colonies.

Competition cultures were inoculated as described above, with the two strains inoculated at the same density. In order to track each strain during competition, the mutant and wild-type strains were marked with either a kanamycin (Kn) resistance gene cassette or a chloramphenicol (Cm) resistance gene cassette inserted into the *lacZ* gene using the method described by Datsenko and Wanner (47). We confirmed that these cassettes themselves did not confer an advantage on cells by competing otherwise wild-type cells with the cassettes against each other and confirming neutral phenotypes (data not shown). Cells were then counted as described above, plating on LB plus Kn (50 μg/ml) or LB plus Cm (50 μg/ml), as appropriate.

### Genomic DNA preparation, sequencing, and analysis.

The DNeasy Blood and Tissue kit (Qiagen) was used to purify genomic DNA from 0.5 ml of culture grown overnight from the previously saved glycerol stocks. Genomic DNA sequencing, single nucleotide polymorphism (SNP) calling, and mutation annotation were performed as described previously with one exception regarding the criterion to call an SNP from the population data (26, 48). Briefly, sequencing libraries were constructed by the Indiana University Center for Genomics and Bioinformatics. Sequencing was performed by the University of New Hampshire Hubbard Center for Genome Studies using the Illumina HiSeq 2500 platform. For each sample, the Illumina reads were aligned to the *E. coli* K-12 (strain MG1655) genome (NCBI reference sequence NC_000913.2) with the short read alignment too, BWA (ver. 0.7.12) (49). The BWA-MEM variant was used with default values for all parameters. For quality control purposes, only unique mappings with a mapping quality score more than 30 and less than 5% mismatch were considered for SNP calling. For each position, an SNP is called if an alternate allele, distinct from the reference allele, is supported by at least three reads from each of the forward and reverse strands (i.e., at least six reads in total). Multiple alternate alleles may be called at the same site if each of them is supported by a sufficient number of reads from both strands, as they may represent different lineages in the population. We also sequenced the parent strain, to ensure that no significant changes from the reference strain were identified. In fact, no changes were identified, even at low diversity, except those already identified as fixed changes from the MG1655 parent (data not shown) (26).

### Site-directed mutagenesis of mutant alleles.

Site-directed mutagenesis was carried out as described in the work of Heermann et al. (38). PFM2^StrR^ was generated by plating an overnight culture of PFM2 on LB plus streptomycin and then restreaked for single colonies. A gene fragment of 355 bp was synthesized to use in the replacement step (Eurofins Genomics). The primers and gene fragments used to generate these alleles can be seen in Table S4 in the supplemental material.

10.1128/mSystems.00192-16.4 TABLE S4  Primer and fragment sequences for point mutation construction. Download TABLE S4, XLSX file, 0.04 MB. Copyright © 2017 Kram et al. 2017 Kram et al. This content is distributed under the terms of the Creative Commons Attribution 4.0 International license.

### Accession number(s).

The sequences reported in this paper have been deposited in the National Center for Biotechnology Information Sequence Read Archive (BioProject accession no. PRJNA362202).
