# Supplementary material for: Adaptation of Escherichia coli to Long-Term Serial Passage in Complex Medium: Evidence of Parallel Evolution
Source: mSystems. 2017 Mar 7;2(2):e00192-16. doi: 10.1128/mSystems.00192-16 (PMC5340864; doi:10.1128/mSystems.00192-16)
Supplement: TABLE S1 [file sys002172093st1.pdf]

Supplemental Table 1. Time points with significant p-values (p ≤ 0.025) compared to unaged cells.

		**Population**								
		**A**			**B**			**C**		
	**Day:**	3	4	5	3	4	5	3	4	5
**Passage**	**3**			0.016	0.019	0.014	0.002	0.013	0.023	0.006
	**6**			0.003	0.021	0.020	0.003	0.012		
	**9**	0.024	0.008	0.004	0.014	0.002		0.013	0.002	
	**12**						0.023	0.014		
	**15**				0.021	0.003	0.012	0.015	0.003	0.017
	**18**		0.012		0.020	0.010			0.005	
	**21**		0.009			0.004		0.014	0.005	0.011
	**24**	0.023	0.005	0.013	0.021	0.009	0.003	0.015	0.008	
	**27**	0.025	0.008		0.019	0.004		0.013	0.014	0.003
	**30**	0.017	0.004		0.021	0.008	0.025	0.021	0.008	0.006
