# Supplementary material for: Adaptation of Escherichia coli to Long-Term Serial Passage in Complex Medium: Evidence of Parallel Evolution
Source: mSystems. 2017 Mar 7;2(2):e00192-16. doi: 10.1128/mSystems.00192-16 (PMC5340864; doi:10.1128/mSystems.00192-16)
Supplement: FIG S1 [file sys002172093sf5.pdf]

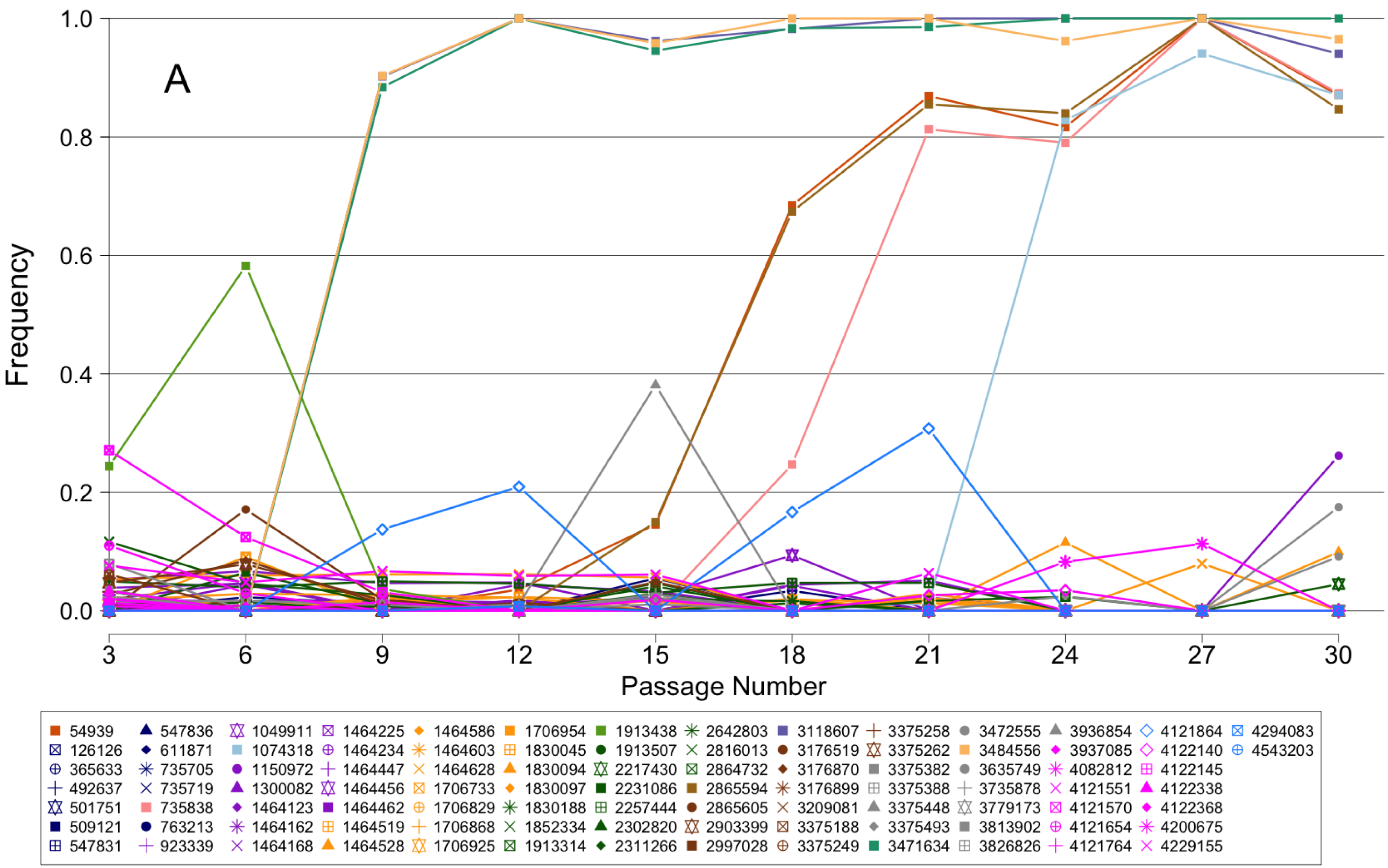

Supplemental Figure 1A. Mutations from passaged populations. Line graph of alleles in each of the three populations for each passage. Frequency was calculated by dividing the number of reads with a non-reference allele by the total number of reads for that nucleotide.

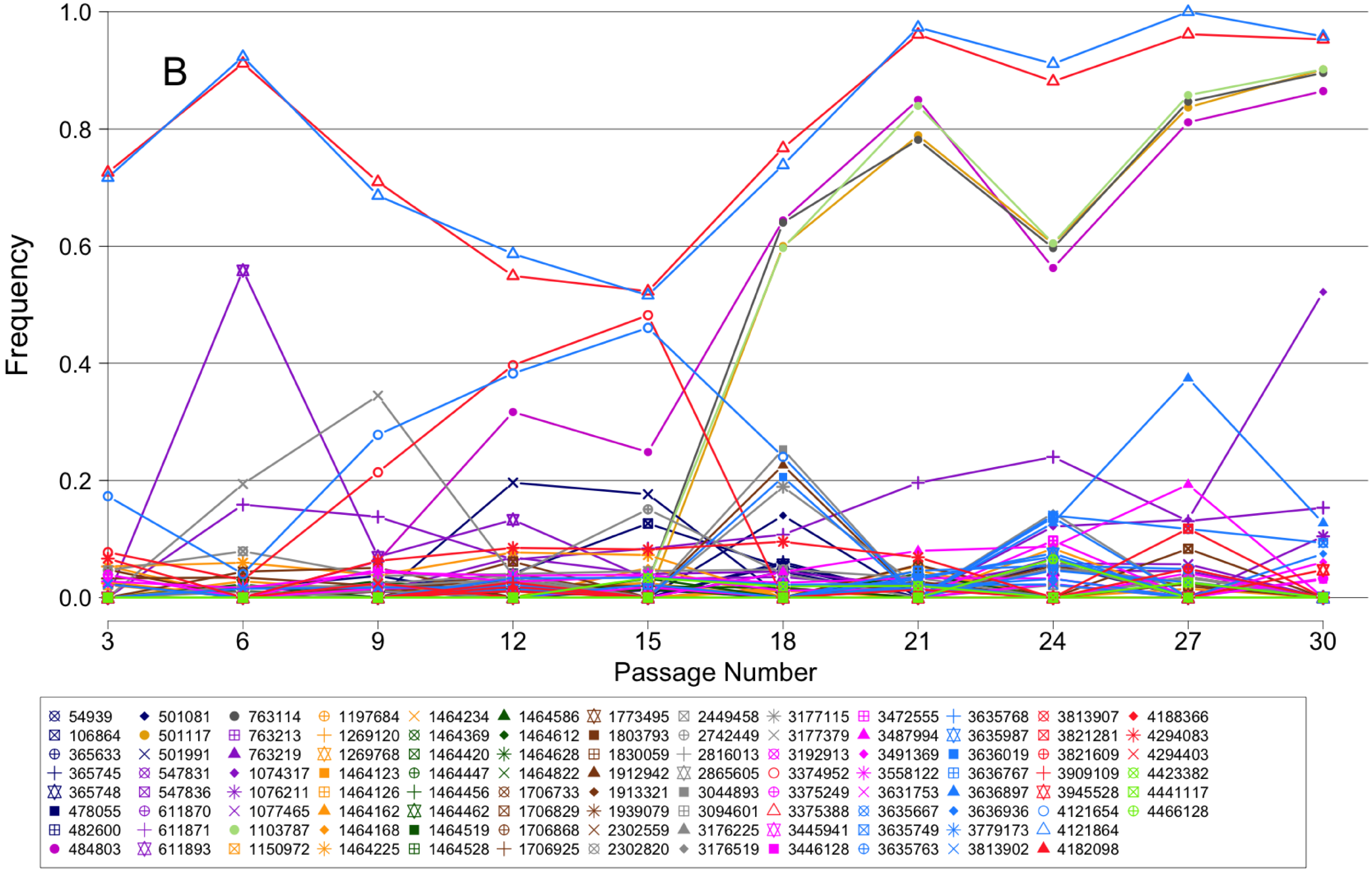

Supplemental Figure 1B. Mutations from passaged populations. Line graph of alleles in each of the three populations for each passage. Frequency was calculated by dividing the number of reads with a non-reference allele by the total number of reads for that nucleotide.

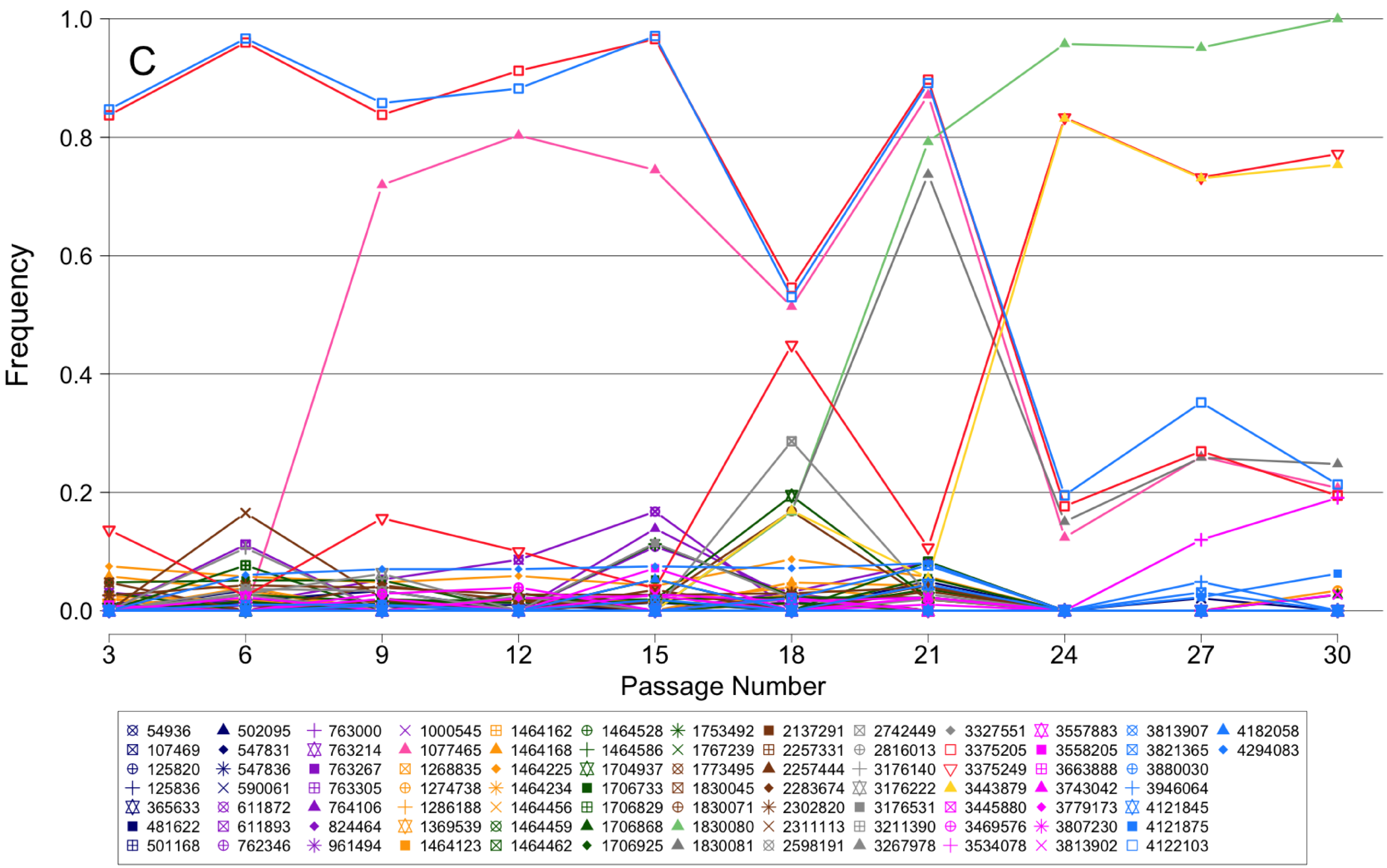

Supplemental Figure 1C. Mutations from passaged populations. Line graph of alleles in each of the three populations for each passage. Frequency was calculated by dividing the number of reads with a non-reference allele by the total number of reads for that nucleotide.
