# Supplementary figures and images for: Adaptation of Escherichia coli to Long-Term Serial Passage in Complex Medium: Evidence of Parallel Evolution
Source: mSystems. 2017 Mar 7;2(2):e00192-16. doi: 10.1128/mSystems.00192-16 (PMC5340864; doi:10.1128/mSystems.00192-16)

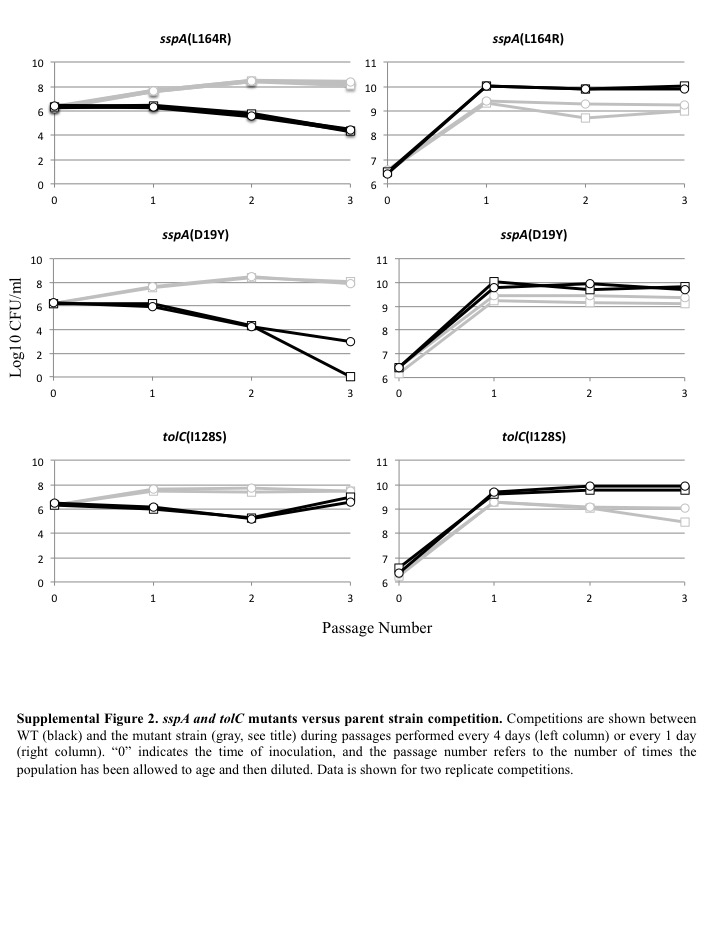

Supplement: FIG S2 [file sys002172093sf6.jpg]
